# External quality assessment for laboratories in pan-India ILI/SARI surveillance for simultaneous detection of influenza virus and SARS-CoV-2

**DOI:** 10.3389/fpubh.2023.1274508

**Published:** 2023-11-13

**Authors:** Varsha Potdar, Neetu Vijay, Veena Vipat, Sheetal Jadhav, Nivedita Gupta, Neeraj Aggarwal

**Affiliations:** ^1^Indian Council of Medical Research–National Institute of Virology, Pune, India; ^2^Division of Epidemiology and Communicable Diseases, Indian Council of Medical Research, New Delhi, India

**Keywords:** EQAP, influenza, SARS-CoV-2, surveillance, ILI, SARI

## Abstract

**Introduction:**

The Indian Council of Medical Research has set up a nationwide network of 28 laboratories for simultaneous surveillance of influenza virus and SARS-CoV-2 in ILI/SARI patients, using an in-house developed and validated multiplex real-time RTPCR assay. The aim of this study was to ensure the quality of testing by these laboratories by implementing an external quality assessment program (EQAP).

**Methods:**

For this EQAP, a proficiency test (PT) panel comprising tissue-culture or egg-grown influenza virus and SARS-CoV-2 was developed. The PT panel was distributed to all the participant laboratories, which tested the panel and submitted the qualitative results online to the EQAP provider. The performance of the laboratories was evaluated on qualitative criteria but cycle threshold (Ct) values were also gathered for each sample.

**Results:**

On a qualitative basis, all the laboratories achieved the criteria of 90% concordance with the results of the PT panel provider. Ct values of different samples across the laboratories were within ≤ ±3 cycles of the corresponding mean values of the respective sample. The results of this EQAP affirmed the quality and reliability of testing being done for simultaneous surveillance of influenza virus and SARS-CoV-2 in India.

## Introduction

Respiratory infections are one of the two infections that are the topmost contributors to global disability-adjusted life years, the other being enteric infections (1). Respiratory infections have a significant impact on public health and the economy (2, 3). Amongst acute respiratory infections, the influenza virus is an important aetiology, especially in children and older adults, accounting for 3 to 5 million cases of severe disease annually (4). The COVID-19 pandemic added another pathogen to this group of infections. Over the last three years, since the advent of Severe Acute Respiratory Syndrome Corona Virus-2 (SARS-CoV-2), more than 656 million confirmed cases with 6.6 million mortalities have been reported globally up to 1 Jan, 2023 (5).

Owing to their RNA genome, both the influenza virus and SARS-CoV-2 are highly prone to mutations, resulting in the evolution of newer strains (6, 7). This characteristic grants both these viruses an ability to cause frequent outbreaks and occasional epidemics or pandemics. Annual outbreaks of influenza are well documented (4). Similarly, the frequent mutations in the SARS-CoV-2 genome have resulted in multiple variants of interest and variants of concern that kept the COVID-19 pandemic ongoing for nearly three years (8). This ability of both these viruses, coupled with a similar clinical picture, emphasizes the need for continuous simultaneous surveillance of these respiratory viruses. The World Health Organization (WHO) in Nov 2020 advised that surveillance for both influenza virus and SARS-CoV-2 be integrated, and the data be reported through the WHO Global Influenza Surveillance and Response System (GISRS) (9). GISRS has been in use for global influenza surveillance since 1952 and has played an important role in the timely detection of globally circulating influenza strains. This platform has facilitated the identification of emerging or reemerging influenza strains. To strengthen the influenza surveillance being done until recently in India in a limited manner by the Indian Council of Medical Research-National Institute of Virology (ICMR-NIV), and to initiate the simultaneous detection of influenza virus and SARS-CoV-2, ICMR established a pan-India surveillance network for influenza virus and SARS-CoV-2 by real-time reverse transcriptase Polymerase Chain Reaction (rRT-PCR) (10). Between 4 July 2021 and 31 October 2022, the network tested 34,260 samples, of which 12.84% samples were positive for one of the two viruses tested along with 37 dual/co-infections (11). Given the multitudinous number of samples being tested by this wide network of laboratories, it is essential to ensure the reliability of testing. This communication describes the external quality assessment program (EQAP) for all the participating laboratories, to establish a quality-assured system for simultaneous qualitative detection and differentiation of influenza virus and SARS-CoV-2 in ILI/SARI patients. EQAP has played a pivotal role in establishing the quality of molecular testing for influenza virus (12) and SARS-CoV-2 (13–15) as individual viruses. To our knowledge, this is the first report on simultaneous EQAP for influenza virus and SARS-CoV-2 virus.

## Methods

### EQAP organization

**Figure 1:**
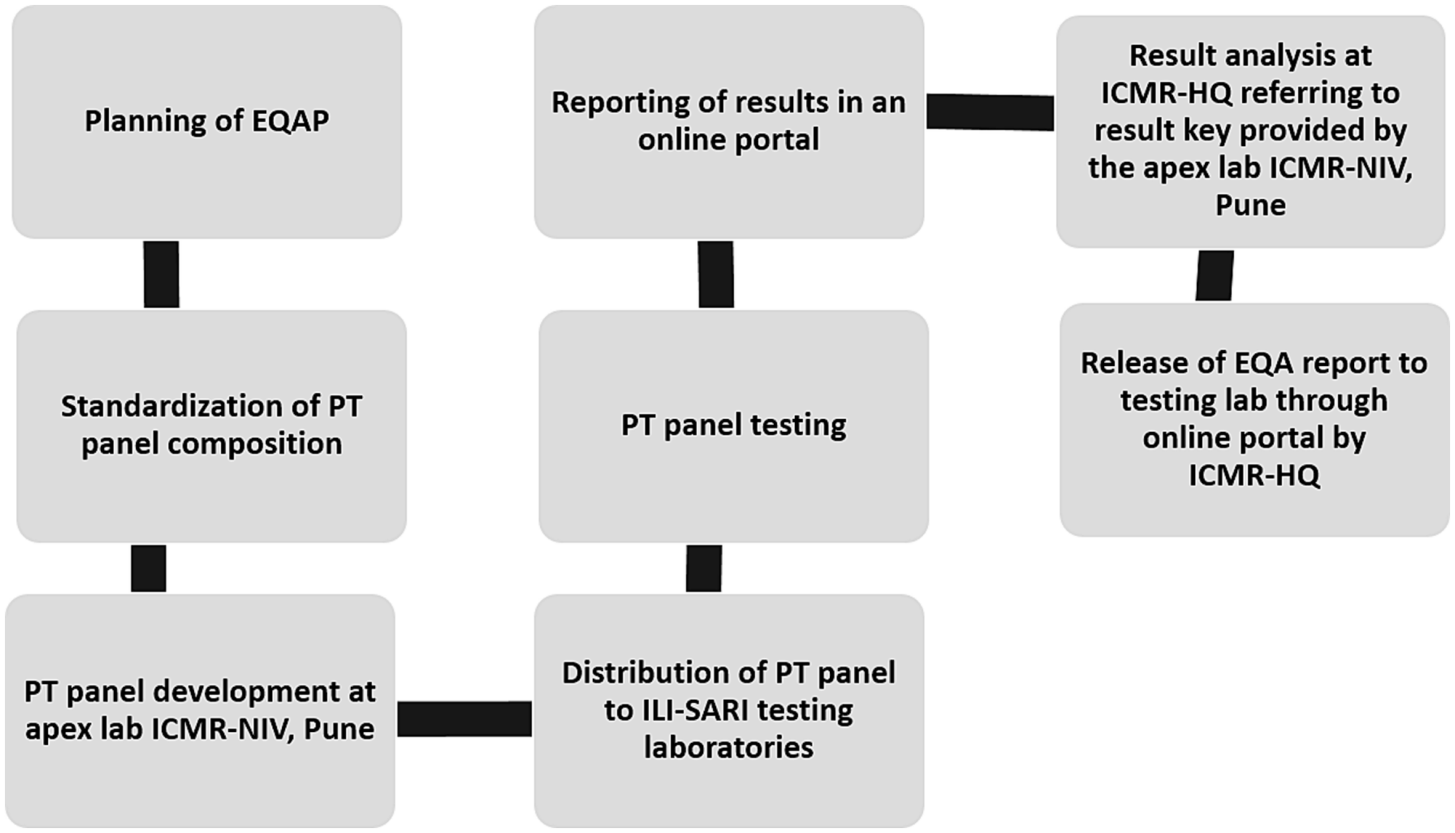
EQAP process flow.

The pan-India surveillance network for influenza virus and SARS-CoV-2 is a three-tiered structure that has 28 laboratories across the length and breadth of the country (10, 11). These laboratories participated in the surveillance of these two respiratory viruses in samples collected from ILI & SARI patients from hospital and defined community settings (11). To ensure the reliability of testing, an EQAP was administered by the ICMR-NIV Pune which is a WHO-NIC for influenza and SARS-CoV-2 reference centre. The program was coordinated by the ICMR Headquarters (ICMR-HQ) to maintain confidentiality and transparency of the results. All testing laboratories barring two participated. The complete flow of the EQAP is depicted in Figure 1.

### Panel composition

EQAP comprised a proficiency test (PT) panel of 12 coded samples. A group of external experts (clinical microbiologists) was constituted by the ICMR-HQ to deliberate on the composition of the PT panel and decide on the scoring and passing criteria. Based on the recommendations of the experts, the PT panel included six contemporary influenza strains, including one avian A(H9N2) virus, two SARS-CoV 2 virus and two negative controls. There were two samples each of influenza A(H1N1)pdm09 and SARS-CoV-2 to represent different viral loads of the same virus type. The panel also included two samples for allelic discrimination by rRT-PCR for testing antiviral susceptibility. The composition of the panel is depicted in Table 1. Each virus sample, except A(H9N2), was an *in-vitro* grown virus whereas A(H9N2) was an egg grown virus. All the viruses were inactivated prior to aliquoting. The PT panel was divided into two sections – section 1, for diagnosis of influenza and SARS CoV 2, comprised of the first ten samples and section 2, for detection of the antiviral susceptibility of A(H1N1)pdm09, comprised of the last two samples. The PT panel was jumbled in different combinations, before shipping out, to avoid any biasness between the laboratories. Each participating laboratory was asked to process samples following the exact procedure that would be used for the patient’s sample. The laboratories had to complete the testing of the PT panel by rRT-PCR using the in-house developed multiplex molecular assay, the testing method used in the pan-India ILI/SARI surveillance network (10), within 10 days from the date of receipt of the PT panel.

**Table 1:** Composition of proficiency test panel used for EQAP.

EQA sample code	Sample type	Virus content
Sample_1	Influenza	Influenza A(H1N1)pdm09
Sample_2	Influenza	Influenza B/Victoria
Sample_3	Negative	Cell culture supernatant
Sample_4	Influenza	Influenza A(H3N2)
Sample_5	SARS-CoV-2	SARS-CoV-2
Sample_6	Influenza	Influenza B/Yamagata
Sample_7	Negative	Cell culture supernatant
Sample_8	Influenza	Influenza A(H1N1)pdm09
Sample_9	SARS-CoV-2	SARS-CoV-2
Sample_10	Influenza	Inf A unsubtypable (Unusual subtype – H9N2)
Sample_11	Influenza	A(H1N1)pdm09 virus wild type (sensitive)
Sample_12	Influenza	A(H1N1)pdm09 virus mutant type (resistant)

### Panel preparation

For preparing the PT panel, early passage Madin-Darby canine kidney (MDCK) cells were infected with previously isolated seasonal influenza viruses. The viruses used for infection had a HA titer of 8–16. After infection, the cells were incubated at 37°C for 3 days in 5% CO_2_ or until the CPE appeared. The culture supernatant was harvested and clarified of cell debris by centrifugation at 10,000 rpm at 4°C. Each virus harvest was antigenically characterized by Haemagglutination assay (HA) and Haemagglutination inhibition assay (HI), as per the protocol described in the WHO manual (16), to determine the HA titer and confirm the subtype of each virus. Once characterized, each virus was heat-inactivated at 50°C for 30 min. The confirmation of complete inactivation was done by three passages of the inactivated virus in MDCK cells. Once confirmed, the viruses were aliquoted in 1 mL aliquots and stored frozen, until shipped, at −80°C. In addition to seasonal influenza viruses, avian influenza A(H9N2) was grown in eggs and gamma irradiated to inactivate the virus (17).

The SARS-CoV-2 (Delta variant B.1.617.2) was received as an inactivated virus from the high containment lab at NIV-Pune (personal communication).

### Result submission and scoring

The key of each combination, based on the results assigned by the apex laboratory ICMR-NIV, was with ICMR-HQ. Each participating laboratory entered results online at the ILI/SARI surveillance portal that is in routine use to capture the surveillance data (10). The ICMR-HQ decoded the results submitted by the laboratories and computed the concordance scores. The scoring was done based only on the results of 10 samples of section one. Each sample was worth a score of 10. For samples no. 1, 2, 4, 6, 8, and 10 (influenza positive), each sample was assessed on two parameters: differentiation of influenza A and influenza B, and identification of subtypes of influenza A or influenza B (each correct reporting fetched a score of 5 each). The sample that had an unusual subtype was to be identified and reported as influenza A un-subtyped. Performance on antiviral susceptibility samples (sample no. 11 and 12) was not considered while computing the scores. These samples were used only to provide feedback on the quality of testing of the laboratory. Each laboratory that scored 90% concordance or more was considered as passing the EQAP.

## Results

This was a qualitative EQAP. Semi-quantitative results (Ct value) were used for reference only.

### Panel characterization at ICMR-NIV

The *in-vitro* propagated isolates of seasonal influenza viruses underwent antigenic characterization. The HA titer of each isolate and their HI titer against respective homologous reference serum is shown in Table 2. Further, each *in-vitro* propagated isolate of seasonal influenza viruses underwent sequencing to check if there have been any changes in genes of the respective viruses due to passage in cell culture. The M and HA gene sequences of the seasonal influenza viruses used in the PT panel aligned completely with the primers and probes used in the multiplex molecular assay, that is used in the pan-India ILI/SARI surveillance network (results not shown), confirming the suitability of the kit for the panel.

**Table 2:** HA and HI titers of *in-vitro* propagated seasonal influenza viruses that were used for PT panel preparation.

Virus subtype	HA titer	HI titer
Influenza A(H1N1)pdm09	1:32	1:320
Influenza A(H3N2)	1:16	1:1280
Influenza B/Yamagata	1:64	320
Influenza B/Victoria	1:64	640

### PT panel results of the testing laboratories

**Figure 2:**
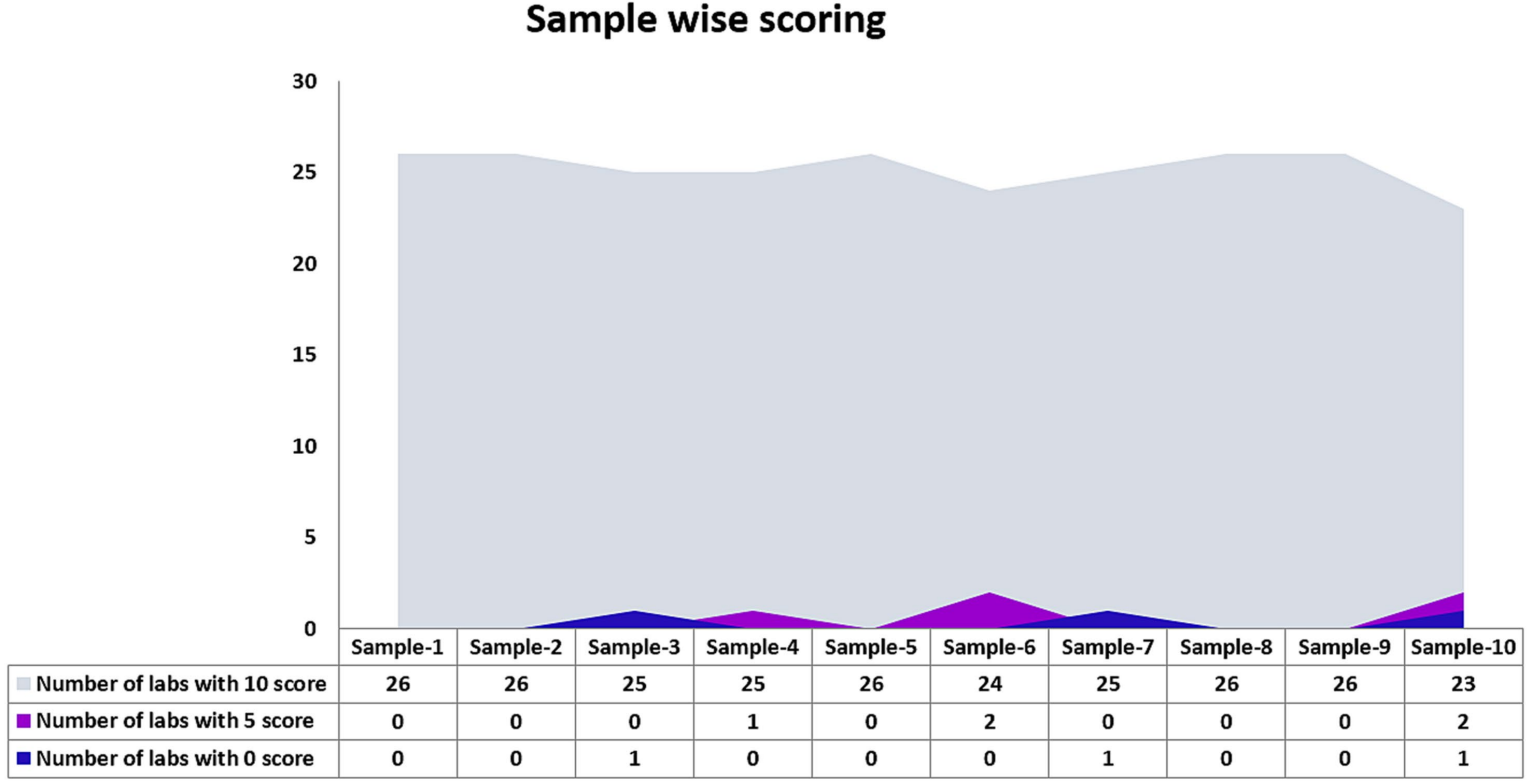
Number of laboratories that detected each sample in section 1 of the PT panel.

**Figure 3:**
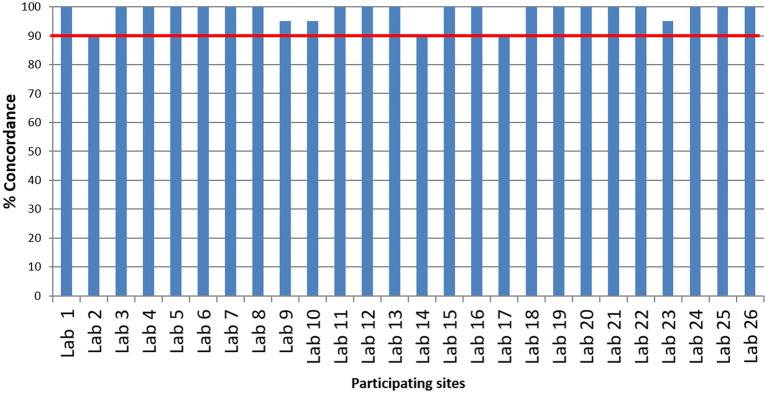
Overall performance of the laboratories.

Twenty-six laboratories that participated in the EQAP tested all the samples in the panel and reported results within the stipulated time. The number of laboratories that detected each sample correctly is shown in Figure 2. Except for one sample by one laboratory, none of the laboratories reported false-negative results and all the laboratories could correctly identify the influenza A, influenza B, or SARS-CoV-2 sample. While subtyping, all the laboratories correctly identified A(H1N1)pdm09 and B/Victoria lineage. A(H3N2) was reported as untypable by one lab and the B/Yamagata lineage was missed by two laboratories. Two laboratories reported influenza A unusual subtype sample as H1N1. False positives were reported by two laboratories (one sample in each lab). Despite these odd reports, all the participating laboratories passed the EQAP by scoring 90% concordance or more (Figure 3).

### Semi-quantitative performance of the laboratories

**Figure 4:**
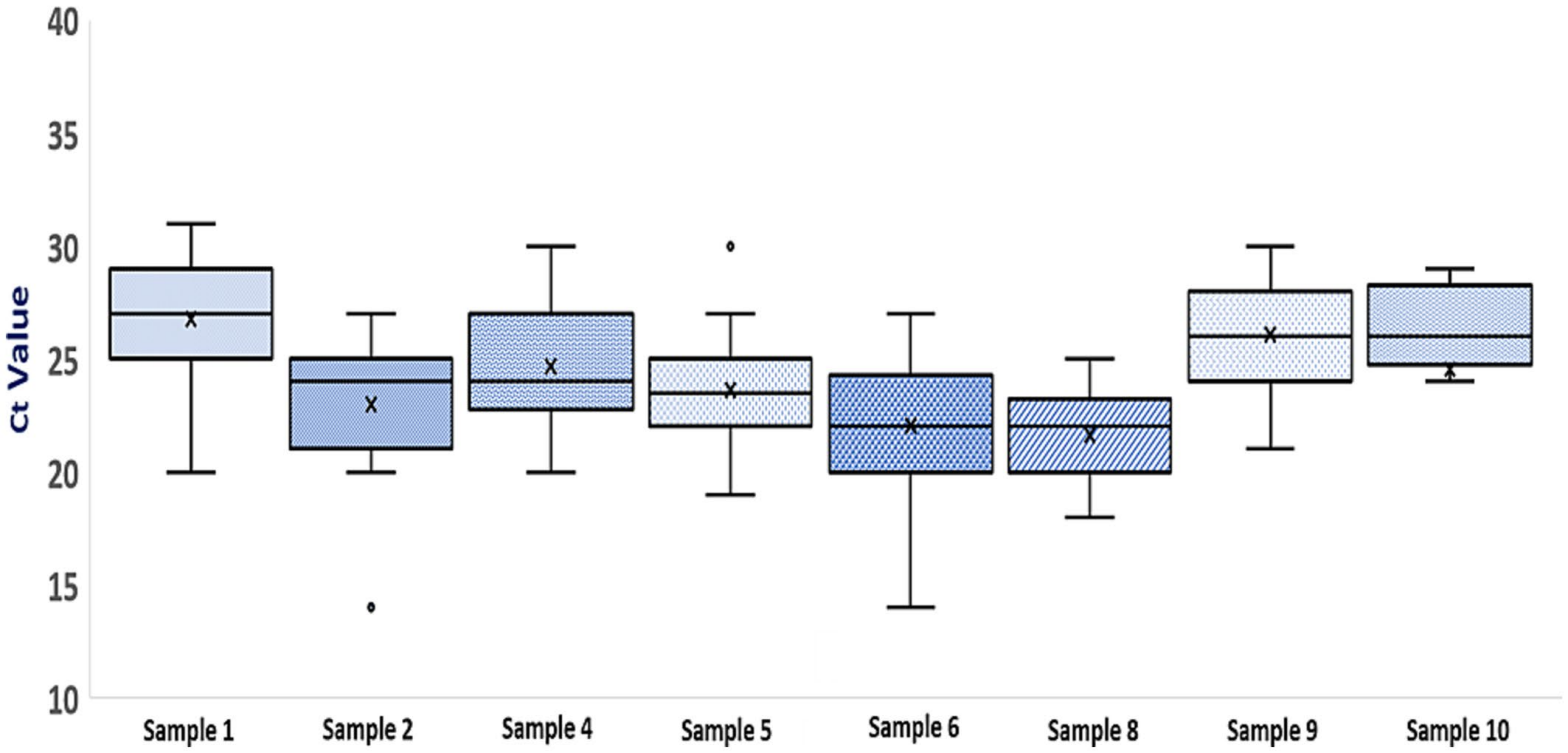
Boxplot of Ct values for samples in section 1 of the PT panel (Each box represents an individual sample. The top and bottom lines of each boxplot represent the third and first quartiles, respectively. The horizontal bar and X within each box represent the median Ct value and mean Ct value for that sample. For each sample, minimum and maximum Ct values are represented by error bars and outliers are represented by O).

Figure 4 shows the Ct value of each sample (except the negative samples) within section 1 of the PT panel for differentiation of influenza A, influenza B, and SARS-CoV-2 samples. Most of the laboratories (73–92%) reported Ct values for individual samples that are within ≤ ±3 cycles of corresponding mean values of the respective sample. When analyzed in terms of interquartile spread, there was an occasional outlier for sample numbers 2 and 5. Nevertheless, most of the laboratories reported Ct values for individual samples that were within the lower and upper quartile of the respective sample. All the laboratories could also discriminate between different viral loads of influenza A(H1N1)pdm09 and SARS-CoV-2, as is seen by the corresponding Ct values, except one lab which failed to do so for high titred SARS-CoV-2 sample (sample number 5).

## Discussion

India initiated surveillance for viral aetiologies of acute respiratory infections by leveraging the network of laboratories that was created under the VRDL scheme (10). The pan-India ILI/SARI surveillance network provided insight into the prevalence of different influenza viruses and SARS-CoV-2 in India over a period of about 15 months, which varied depending on the time of the year and seasonality (11). Given the numerous number of tests being done, it was imperative to gain confidence in the reliability of the test results. EQAP, through PT panel testing, is a time-tested method in laboratory medicine to address discrepancies amongst laboratories when the same analyte is measured by the same method (18). EQAP permits simultaneous testing of multiple samples that vary from negative to strong positive samples. This allows covering the entire spectrum of samples that a laboratory can potentially test. Furthermore, EQAP is run in a coded manner where neither the panel composition nor the probable results are known to the participating laboratories. This simulates the everyday operations of the lab. Independent third-party monitoring of results reported by the testing laboratories, in comparison to the results reported by the EQAP provider, enhances the confidence of all the participating laboratories in the process. EQAP is a tool that has been extensively used as a measure of the quality of testing in multiple test methods (14, 15, 19–23) including molecular testing for influenza virus and SARS-CoV-2 (12, 13). EQAP for influenza virus and SARS-CoV-2 have been standalone activities (12–15) and there has been no published report on a simultaneous EQAP for both these viruses. Therefore, an EQAP was developed and administered by the national reference laboratory at ICMR-NIV Pune to ascertain the quality of integrated testing for influenza virus and SARS-CoV-2 in the pan-India ILI/SARI surveillance network.

Qualitatively, the performance of all the participating laboratories was satisfactory as they all met the qualifying criteria. The qualitative results when analyzed semi-quantitatively, showed small discrepancies in Ct values for the same sample amongst laboratories. Nevertheless, the laboratories could correctly discriminate different viral loads of the same virus (sample 1 vs. 8 and sample 5 vs. 9). Variation in Ct values for the same sample across laboratories has been reported in other EQAPs too (14, 24, 25). Variation in Ct could be attributed to the lack of adherence to good laboratory practices such as the use of standardized test protocol, regular calibration of equipment, and technical ability of staff familiar with the protocol. Such practices are known to impact the performance of laboratories. The performance of the laboratories that follow CLIA regulations in the U.S.A. has been shown to be better (26), thus emphasizing the need to follow certain minimum standards. Caution is advised while relying on Ct values for patient management. However, some clinicians opine that Ct values are helpful in decision-making (27) and thus request laboratories to report Ct values. For reporting comparable Ct values across the pan-India network, laboratories are advised to locally standardize and validate the test protocol and emphasis needs to be laid on the calibration of test equipment. A limitation of our PT panel was that the panel comprised of cell-culture or egg-grown viruses and thus did not represent an actual clinical sample. We resorted to this strategy due to the lack of availability of clinical samples in sufficient volumes. Our panel could not accommodate samples of all the viruses that represented high, medium, and low viral loads due to logistics issues. Therefore, we used two samples with varying viral load and the rest of the samples had medium viral load, for a fair performance assessment of the laboratories. Another limitation was that no reference standard was included in the panel to address the standardization of the test protocol. The EQAP indicated that integrated influenza virus and SARS-CoV-2 diagnosis, on a qualitative level, by all the laboratories participating in the pan-India ILI/SARI surveillance network is accurate and the in-house developed multiplex molecular assay performs reliably. From an epidemiological perspective, this is significant as co-circulation of influenza and SARS-CoV-2 has been documented in India (10). Thus, it becomes important that assays in use detect the underlying pathogen, accurately and reliably, without raising false positives. To our knowledge, this is the first national-level EQAP to assess the capability of testing laboratories to simultaneously detect influenza virus and SARS-CoV-2 using a multiplex rRT-PCR.

## Data availability statement

The original contributions presented in the study are included in the article/Supplementary material, further inquiries can be directed to the corresponding author.

## Author contributions

VP: Methodology, Resources, Writing – review & editing. NV: Conceptualization, Data curation, Formal analysis, Funding acquisition, Investigation, Project administration, Writing – review & editing. SJ: Methodology, Resources, Validation, Writing – review & editing. VV: Methodology, Resources, Writing – review & editing. NG: Conceptualization, Supervision, Writing – review & editing. NA: Conceptualization, Supervision, Writing – original draft.
