# Supplementary figures and images for: External quality assessment for laboratories in pan-India ILI/SARI surveillance for simultaneous detection of influenza virus and SARS-CoV-2
Source: Front Public Health. 2023 Nov 13;11:1274508. doi: 10.3389/fpubh.2023.1274508 (PMC10679675; doi:10.3389/fpubh.2023.1274508)

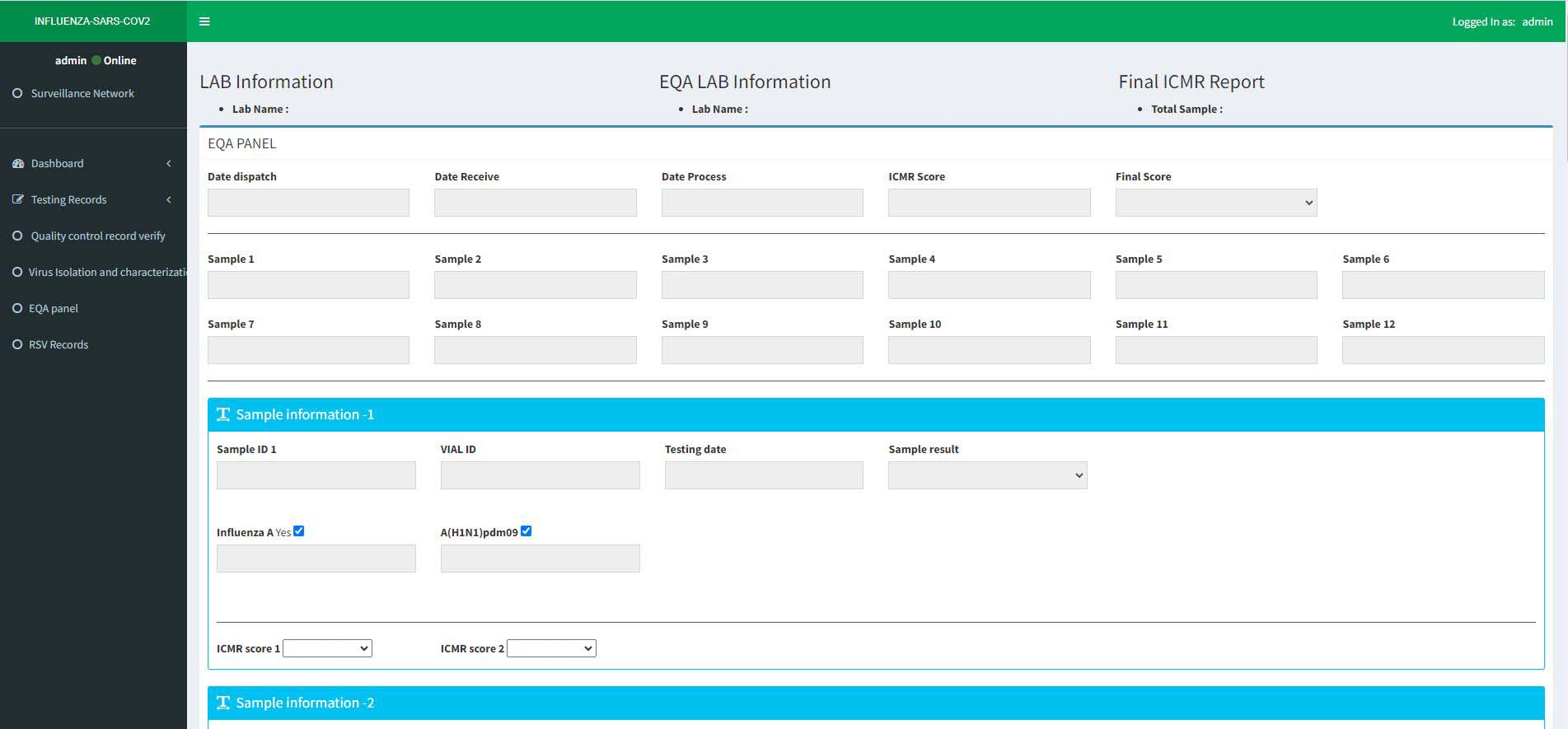

Supplement: Supplementary file 2 [file Image_1.JPEG]
